# The Max b-HLH-LZ Can Transduce into Cells and Inhibit c-Myc Transcriptional Activities

**DOI:** 10.1371/journal.pone.0032172

**Published:** 2012-02-22

**Authors:** Martin Montagne, Nicolas Beaudoin, David Fortin, Christine L. Lavoie, Roscoe Klinck, Pierre Lavigne

**Affiliations:** 1 Département de Pharmacologie, Faculté de médicine et des sciences de la santé, Université de Sherbrooke, Québec, Canada; 2 Département de Chirurgie, Faculté de médicine et des sciences de la santé, Université de Sherbrooke, Québec, Canada; 3 Département de Microbiologie et Infectiologie et Laboratoire de Génomique Fonctionnelle de l'Université de Sherbrooke, Québec, Canada; Ottawa Hospital Research Institute, Canada

## Abstract

The inhibition of the functions of c-Myc (endogenous and oncogenic) was recently shown to provide a spectacular therapeutic index in cancer mouse models, with complete tumor regression and minimal side-effects in normal tissues. This was achieved by the systemic and conditional expression of *omomyc*, the cDNA of a designed mutant of the b-HLH-LZ of c-Myc named Omomyc. The overall mode of action of Omomyc consists in the sequestration of Max and the concomitant competition of the Omomyc/Max complex with the endogenous c-Myc/Max heterodimer. This leads to the inhibition of the transactivation of Myc target genes involved in proliferation and metabolism. While this body of work has provided extraordinary insights to guide the future development of new cancer therapies that target c-Myc, Omomyc itself is not a therapeutic agent. In this context, we sought to exploit the use of a b-HLH-LZ to inhibit c-Myc in a cancer cell line in a more direct fashion. We demonstrate that the b-HLH-LZ domain of Max (Max*) behaves as a *bona fide* protein transduction domain (PTD) that can efficiently transduce across cellular membrane via through endocytosis and translocate to the nucleus. In addition, we show that the treatment of HeLa cells with Max* leads to a reduction of metabolism and proliferation rate. Accordingly, we observe a decrease of the population of HeLa cells in S phase, an accumulation in G1/G0 and the induction of apoptosis. In agreement with these phenotypic changes, we show by q-RT-PCR that the treatment of HeLa cells with Max* leads to the activation of the transcription c-Myc repressed genes as well as the repression of the expression of c-Myc activated genes. In addition to the novel discovery that the Max b-HLH-LZ is a PTD, our findings open up new avenues and strategies for the direct inhibition of c-Myc with b-HLH-LZ analogs.

## Introduction

c-Myc and Max are members of a large network of basic region-Helix-Loop-Helix-Leucine Zipper (b-HLH-LZ) transcription factors. This network also includes L-Myc, N-Myc and the proteins from the Mad family (Mad1, Mxi1, Mad3 and Mad4). The Myc and the Mad proteins exert their transcriptional activities as Myc/Max and Mad/Max heterodimers [1],[2],[3],[4],[5],[6],[7]. Max is the only protein in the network able to homodimerize. It is the HLH-LZ domains that are responsible for the homodimerization of Max and the specific heterodimerization with Myc and Mad proteins, while the basic regions mediate the specific DNA binding [4]–[9]. All the dimers of this network bind a specific DNA sequence called the consensus E-Box (CANNTG) located in the promoters of c-Myc target genes [4],[5],[6],[7],[8],[9]. Once bound to the E-Boxes at core promoters, c-Myc recruits, through its transactivation domain (TAD), co-activators (TRAPP and GCN5) with Histone Acetyl Transferase (HAT) activities. While Max does not possess a specialized domain capable of recruiting co-repressors, its overexpression has been shown to inhibit c-Myc induced proliferation through the competition for E-box sequence at target gene promoters [10], [11]. Accordingly, the overexpression of Max was shown not to be oncogenic because of the high levels of expression compared to those of c-Myc [11].

The most recent estimates indicate that c-Myc regulates (up and down) the transcription of up to 15% of the genome [12],[13]. Genes that are activated are generally involved in the cell cycle progression (proliferation) and metabolism (growth), whereas the list of repressed genes contains cell cycle inhibitors. Indeed, c-Myc activates genes such CDKs and cyclins, ribosomal RNAs and proteins implicated in the ribosomal genesis (e.g. nucleolin). p15Ink4b, p21cip1 and p27kip1 are amongst the key repressed genes.

In comparison to the mechanism of transactivation, the mode of repression by c-Myc is less well understood [14]. It has been demonstrated that c-Myc, as a heterodimer with Max, cooperates with Miz1 to repress transcription of cell cycle inhibitors such as p15, p21 and p27 [2],[4],[15],[16],[17],[18],[19],[20]. The recruitment of c-Myc to the p15 and p21 core promoters is thought to be solely dependent on DNA bound Miz1 [15],[16],[17]. However, it has been recently reported that direct binding to E-box sequences located at repressed promoters also contributes to the repression mechanism of p15 and p21. Since Max does not interact with Miz1, it is possible that the anti-proliferative effect observed with its overexpression could involve the activation of the transcription of cell cycle inhibitors and the repression of the transcription of pro-proliferative genes.

c-Myc is overexpressed in the majority of malignancies, and while its role in the tumorigenesis of these lesions has been clearly established, the pertinence of targeting c-Myc as a therapeutic treatment has been debated. Indeed, because c-Myc is involved in so many aspects of cell growth and proliferation, inhibiting its functions could cause serious side-effects in normal tissues. However, Soucek and Evan [21], have shown that the systemic expression of *omomyc* - the cDNA coding for a mutated form of the b-HLH-LZ of c-Myc called Omomyc [22],[23], [24] – leads to clearance of tumors in mouse models of lung and pancreatic islet tumors without causing damage to normal tissues. These results demonstrated, for the first time, that c-Myc is a valid therapeutic target and that its systemic inhibition can provide a high therapeutic index. The mode of action of c-Myc resides in the repression of c-Myc activated transcription alone. This is achieved through the competition between the Omomyc/Max complex with the endogenous c-Myc/Max heterodimer for E-box sequences at activated core promoters. However, the expression of *omomyc* does not activate c-Myc repressed transcription. This is explained by the fact that the Omomyc can still interact with Miz1 at repressed core promoters.

While Omomyc has provided invaluable insights to understand and model the validity of targeting c-Myc as a therapeutic approach and how a b-HLHLZ domain from the c-Myc/Max/Mad can be used to inhibit c-Myc, it is not a therapeutic agent *per se*. However a b-HLH-LZ that could spontaneously and autonomously transduce (penetrate) inside cells, translocate to the nuclei and inhibit c-Myc transcriptional activities would certainly represent a considerable step towards the development of a cancer therapeutic agent.

In this study, we show that the b-HLH-LZ domain of Max (Max*) possesses the ability to transduce into HeLa cells, translocate to the nucleus and interfere with the transcriptional activities of c-Myc. We show that the treatment of HeLa cells with Max* leads to the transcriptional repression of pro-proliferative genes and the transcriptional activation of cell cycle inhibitors. Accordingly, HeLa cells are observed to undergo a G1 arrest and apoptosis when treated with Max*. A substantial reduction in the metabolism of treated HeLa cells is also observed. In summary, our study reveals that the b-HLH-LZ of Max is a *bona fide* PTD with the ability to inhibit c-Myc in cancer cells. These findings are of fundamental importance for the development therapeutic b-HLH-LZ domains designed to inhibit c-Myc.

## Results

### Max* can efficiently transduce across HeLa cell membranes by endocytosis

**Figure 1:**
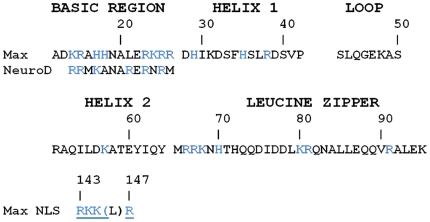
The b-HLH-LZ of Max has a putative NLS sequence and PTD in its basic region. Primary structure of the b-HLH-LZ of Max (Max*). Also shown is the domain of NeuroD responsible for its transduction and nuclear localization and the Max NLS. Note the high content in basic side-chains and the similarity between the sequences.

**Figure 2:**
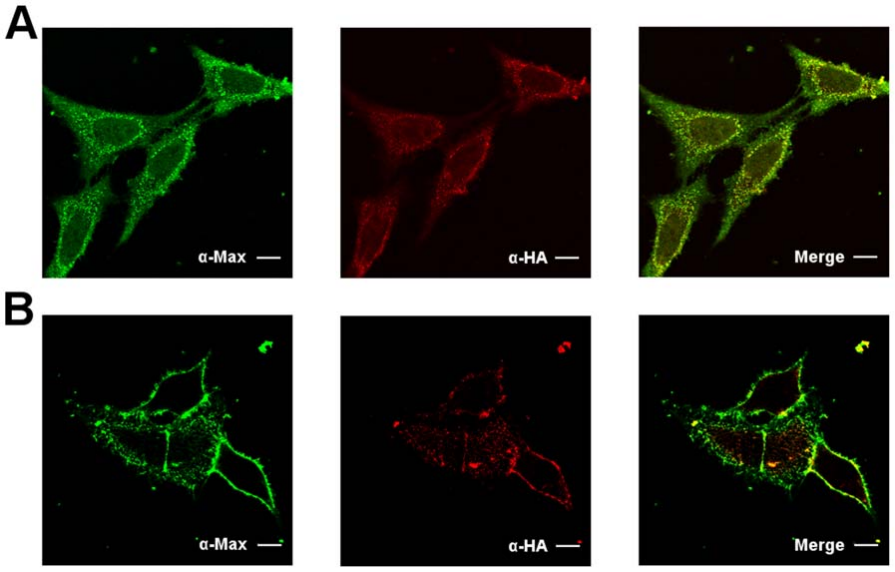
The b-HLH-LZ of Max directly transduces into HeLa cells. Confocal immunofluorescence photomicrographs of HeLa cells incubated for 20 minutes with Max*-HA at 37°C A) and 4°C B). Detection with α-Max, α-HA tag and the merge are displayed from left to right respectively. A) Note the punctuated (endosomes) and diffused labeling inside the cytoplasm and nucleus. B) At 4°C, internalization is inhibited and the fluorescence is confined to the cell surface. Note the colocalization of the fluorescence of the α-Max and α-HA labeling. Scale bar = 10 µm.

It has been recently shown that NeuroD, a b-HLH transcription factor, can spontaneously transduce into cells, translocate into their nuclei and elicit transcriptional regulation [25]. The peptide sequence, RRMKANARERNRM, located in the basic region was shown to be responsible for the transduction (micropinocytosis) and the nuclear localisation of NeuroD. As shown in Figure 1, a homologous sequence is located in the basic region of Max. This region is also similar to a nuclear localization signal (NLS) located at the C-terminus of Max [26]. These similarities prompted us to test, by immunofluorescence and confocal microscopy, whether the b-HLH-LZ of Max (Max*) could also behave as a PTD and translocate into the nucleus of HeLa cells (Figure 2).

**Figure 3:**
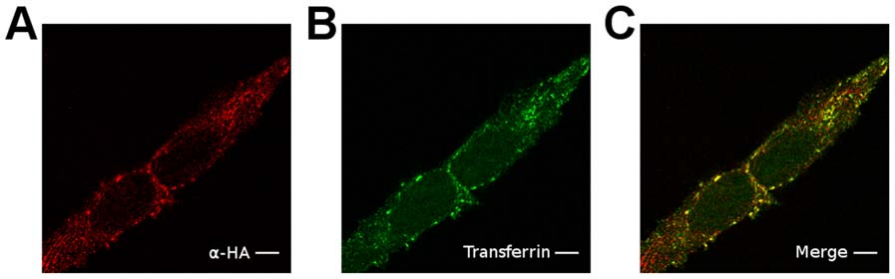
The transduction of Max* colocalizes with the internalized Transferrin. Confocal photomicrographs of HeLa cells simultaneously incubated with Max*HA and Transferrin-^AF488^ for 30 minutes. A) Immumofluorescence labeling of Max*HA, B) Fluorescence of Transferrin-^AF488^ and C) Merge. Scale bar = 10 µm.

In order to discriminate between Max* and the endogenous Max, a HA tag was fused to the end of the LZ of Max* to generate the Max*HA construct. Since the conditions (antibody concentration and laser power) were set to give no endogenous Max background, the transduction phenomenon should be revealed by both the α-Max (polyclonal) and α-HA. As shown in Figure 2A, a 20 minute incubation of HeLa cells with 10 µM of Max*HA led to diffuse (cytoplasm and nucleus) and punctuated intracellular labeling. As expected, the diffuse and punctuated immunofluorescence of the Max and HA antibodies are clearly colocalized. The same diffuse and punctuated immunofluorescence is obtained when HeLa cells are treated with Max* without the HA tag and the latter is revealed with the monoclonal α-Max (*vide infra*). This result indicates that the HA tag is not responsible for the transduction and that it is dispensable for the discrimination of endogenous Max and exogenous Max*. The cytoplasmic inclusions (punctuated labeling) observed in Figure 2A resembled to endosomal structures. These could originate from macropinocytosis or receptor-mediated endocytic uptake. In order to unveil the actual transduction mechanism, cells were treated and incubated at 4°C to inhibit internalization. At this temperature, the immunofluorescence is confined to the membrane with little or no detectable signal in the cytoplasm and the nucleus (Figure 2B). Since cellular uptake and/or translocation is affected by temperature [27]. This result supports the notion that Max*HA transduces through an active cellular uptake such as endocytosis. To confirm that Max*HA transduces by endocytosis, HeLa cells were simultaneously treated with Max*HA and Transferrin-^AF488^, a *bona fide* ligand of the clathrin-dependant endocytic pathway. As displayed in Figure 3, Max*HA (Fig. 3A) and Transferrin-^AF488^ (Fig. 3B) are colocalized (Fig. 3C) in similar and common structures. Although partial, this co-localization further indicates a clathrin-dependent uptake of Max*HA.

**Figure 4:**
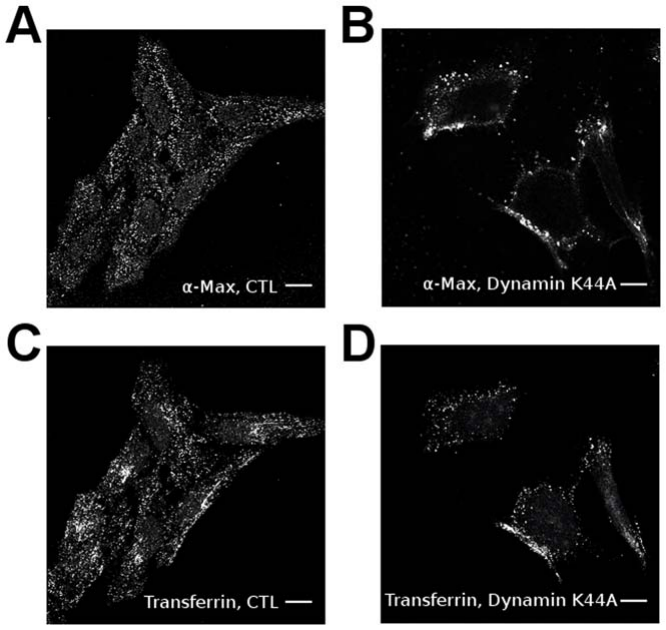
The transfection of the defective *Dynamin1* K44A mutant blocks the endocytosis of Max* into HeLa cells. Confocal photomicrographs of HeLa cells transfected or not with a vector encoding for the dominant-negative mutant of *dynamin1*(K44A) and incubated with Max* and AlexaFluor-488 transferrin for 20 minutes at 37°C (subsequent to a 30 minutes pre-incubation period at 4°C). Immunofluorescence staining of Max* without A) and with B) the transfection of the Dynamin1 (K44A) mutant. Fluorescence of Transferrin-^AF488^ without C) and with D) the transfection of the Dynamin1 (K44A) mutant. Scale bar = 10 µm.

**Figure 5:**
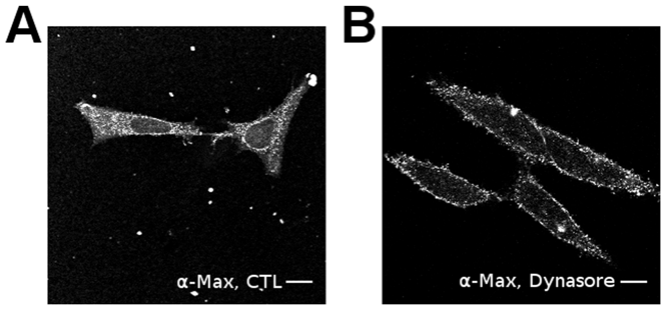
The transduction of Max* in HeLa is blocked by Dynasore. Confocal immunofluorescence photomicrographs of HeLa cells incubated with Max* for 20 minutes at 37°C (subsequent to a 30 minutes pre-incubation period at 4°C) in the absence A) or presence of B) 25 µM Dynasore. Scale bar = 10 µm.

Clathrin-dependent endocytosis is dependent on Dynamin1 [28]. Dynamin1 is a GTPase implicated in the scission of the new vesicles from the cytoplasmic membranes. To verify if Max* is transduced by a Dynamin-dependent endocytosis, we transiently transfected HeLa cells with a dominant negative and GTPase defective Dynamin1 (K44A mutant) [29], [30] prior incubation with Max* for 30 minutes. As one can observe in Figure 4, the transient transfection of the dominant negative effectively inhibits the endocytosis of Transferrin-^AF488^ as well as Max*. Indeed, both proteins are at the cell surface or in vesicles near the plasma membrane. As stated above, this experiment also highlights the ability of Max* to transduce into HeLa without the necessity of the HA tag. In order to provide additional credence to the dependence of the transduction of Max* on Dynamin1, we treated cells with the Dynamin inhibitor, Dynasore [31], prior to incubation with Max* (Figure 5). As can be seen, the endocytosis of Max* is blocked by the Dynasore treatment. Taken together these experiments demonstrate that Max* can transduce into HeLa cells through a clathrin- and Dynamin-mediated endocytic mechanism.

### Time dependent cellular distribution and nuclear accumulation

**Figure 6:**
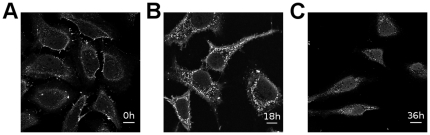
Accumulation of Max* in the nucleus of HeLa cells. Confocal immunofluorescence photomicrographs of HeLa cells incubated with Max* for 12 hours, washed and chased for 0 hour (A) 18 hours (B) and 36 hours (C). Cells were fixed, permeabilized and immunolabeled with anti-Max antibodies. Scale bar = 10 µm.

In order to follow the intracellular transport of Max*, HeLa cells were incubated with Max* for 12 hours at 37°C. Cells were then carefully washed and the immunofluorescence was subsequently monitored after 0, 18 and 36 hours (Figure 6A, B and C). All the laser settings and concentrations were kept constant in order to evaluate the relative distribution of the fluorescence. One can see a gradual accumulation of a diffuse nuclear fluorescence with time. Concomitantly, a decrease in the cytoplasmic diffused and punctuated fluorescence is also observed. This is suggestive of endosomal escape of Max* and a net flux into the nucleus. However, the persistence of the punctuated fluorescence implies that the endosomal escape is not total. These results indicate a time dependent accumulation of Max* into the nuclei of HeLa cells.

### The treatment of HeLa cells with Max* leads to the inhibition of c-Myc transcriptional activities

**Figure 7:**
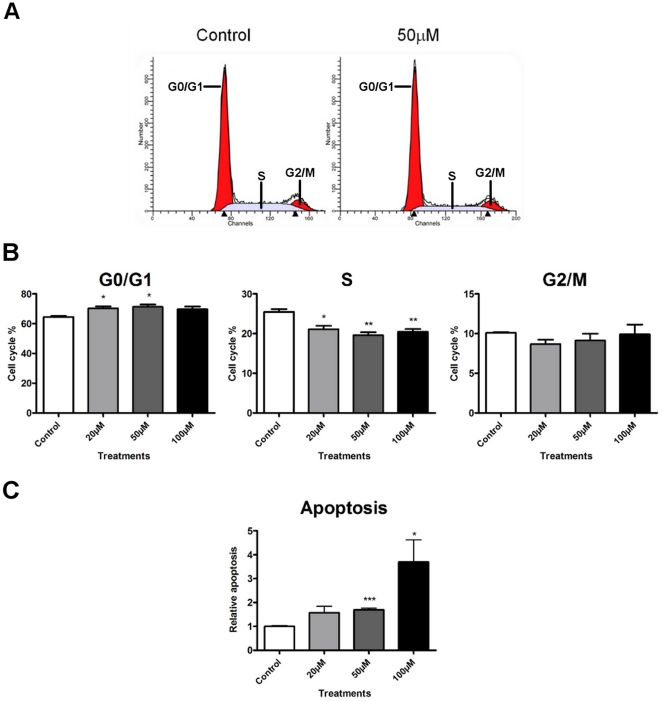
The treatment of HeLa cells with Max* induces cell cycle arrest and apoptosis. A) Representative cell cycle distribution determined by flow cytometry (FACS) and propidium iodide (PI) labeling with (right) and without (left) treatment (50 µM, 72 hours) with Max*. B) Histogram representation of the populations of HeLa cells in each phase as a function of treatment concentration. C) Relative level of apoptotic cells as determined by sub-G1 events. N = 3, * P<0.05, ** P<0.01 and *** P<0.0001.

To validate the expected inhibitory effect of a Max* treatment on c-Myc transcriptional activities in HeLa cells, we monitored the mRNA levels of key target genes repressed and activated by c-Myc by qPCR. As summarized in Figure 7A, a dose-dependent and transient up-regulation of p21 (Figure 7B) and p27 is observed at 24 hours. At 48 hours, the transcription of Rb1 is also upregulated. In accordance with our expectations, a dose-dependent repression of CyclinD1 (Figure 7C) and CyclinB1 as well as cdc25a (24 hours), CDK4 (48–72 hours) and CyclinE1 (72 hours) is observed.

### Cell cycle analysis and apoptosis of HeLa cells treated with Max*

**Figure 8:**
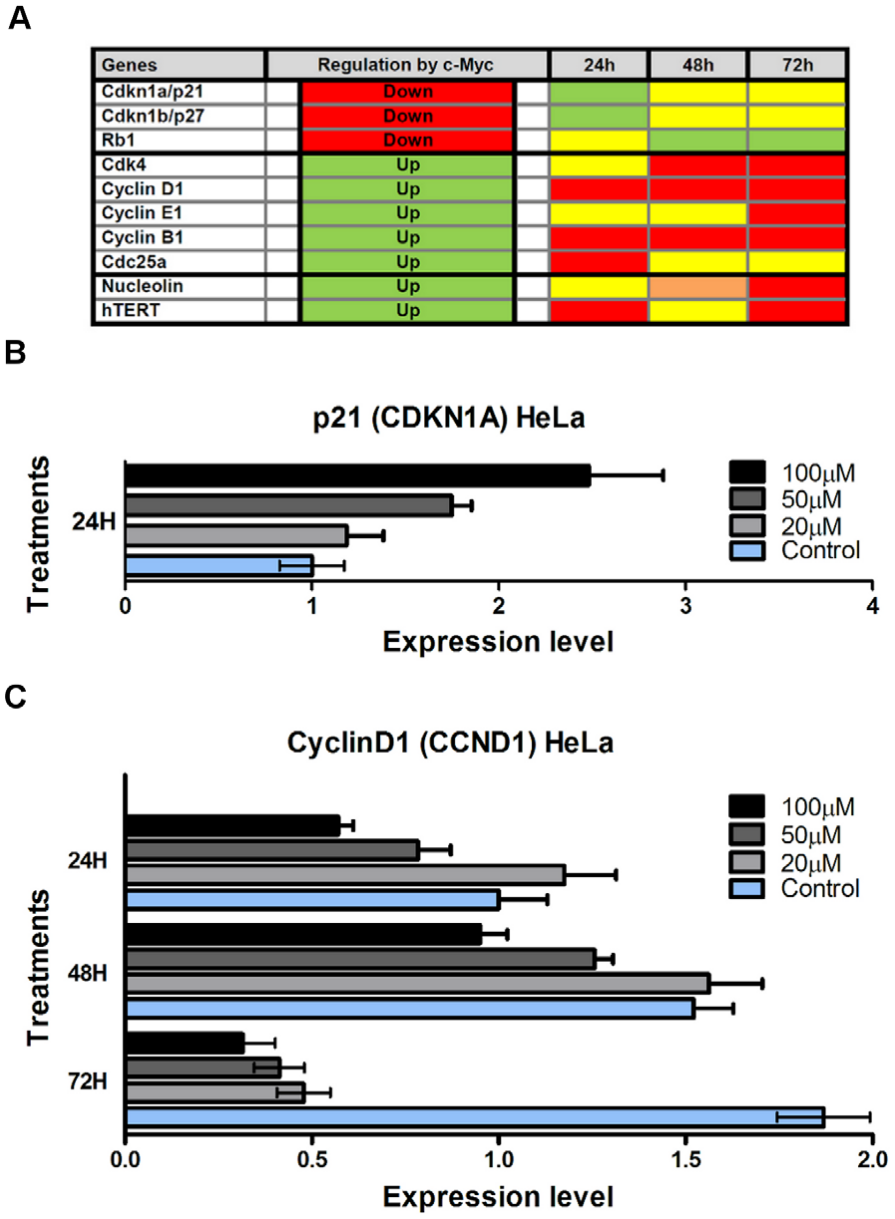
Max* inhibits c-Myc transactivation and repression of transcription. A) Table summarizing qPCR measurement of the effect of a treatment with Max* on a subset of repressed (second column, red) and activated (second column, green) c-Myc target genes in HeLa cells. Green and red boxes indicate statistically significant dose-dependent activation and repression in transcription, respectively. Yellow boxes indicate no statistically significant trend. Orange boxes indicate non-statistically significant repression. Representative results are shown in B) and C) for activated (p21) and repressed (Cyclin D1) genes by Max*, respectively.

Given that an upregulation of p21, p27 and Rb1, coupled to a downregulation of Cyclins B1, D1and E1 and CDK4 should, classically, give rise to a G1 arrest, we investigated the effect of Max* treatment on the cell cycle distribution of HeLa cells by FACS. As shown in Figures 8A, B and C, the treatment of HeLa cells with 50 µM Max* leads to a significant increase in the population of cells in G0/G1 and a decrease in the population of cells in the S phase. Concurrently, no significant accumulation of cells in G2/M was detected (Figure 8D). However as shown in Figure 8E, a significant percentage of cells are undergoing apoptosis.

### Inhibition of the proliferation of HeLa cells by Max* treatment

**Figure 9:**
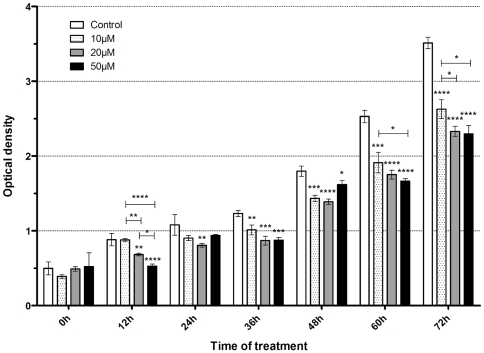
Evaluation of the expansion of viable HeLa cells with WST-1 assays. Cells were treated with different doses of Max* (hatched bars, 10 µM; grey bars, 20 µM and black bars 50 µM) for a period up to 72 hours before the WST-1 assay. Note the dose and time dependent reduction of expansion compared to the control (white bar). *, p<0.05; **, p<0.001; ***, p<0.0001; ****, p<0.00001.

The observed reduction of the population of HeLa cells in S phase and the accumulation in G1 phase due to the treatment with Max* should also lead to a reduction in proliferation, or in the expansion of viable cells. To evaluate the impact of Max* treatment on the proliferation of HeLa cells we used a colorimetric WST-1 cell expansion assay. As shown in Figure 9, treatment with increasing concentrations of Max* decreases cell expansion in a dose-dependent manner. A 34.8% decrease in proliferation is observed at 72 hours with a single treatment with 50 µM of Max*.

## Discussion

In this work, we have demonstrated for the first time the ability of the b-HLH-LZ domain of Max (Max*) to transduce into HeLa cells by a Dynamin-dependent endocytic mechanism (Figures 2, 3, 4, and 5). Although not complete, we have found that Max* was able to escape endosomes and to accumulate in the nucleus of HeLa cells (Figure 6). Similar results have been obtained with HCT116, F98, U118 and U87 cell lines (data not shown) indicating that this property is not specific to HeLa cells. Although we have not demonstrated which domains of Max* are responsible for the transduction and nuclear localization, it is interesting to note that Max* shares a sequence in its basic region which is highly homologous to that of NeuroD (a b-HLH transcription factor), also located in its basic region, which was demonstrated to be responsible for its transduction and nuclear localization (Figure 1). Hence, it is tempting to suggest that this sequence, KRAHHNALERKRR, is also responsible for both activities. Consistently, its high basic residue content (K and R) meets the requirement for the existence of a PTD sequence [32]. Moreover, a NLS in the C-terminus of Max with the following sequence RKKLR has been identified [26]. This sequence is also highly similar to the C-terminal part of the putative PTD/NLS sequence identified in Max* (Figure 1). Work is underway in our laboratory to identify the domains of Max* responsible for its internalization and nuclear localization. Current and future efforts also aim to improve these activities and optimize the endosomal escape.

We have also shown that the incubation of HeLa cells with Max* considerably decreased cellular proliferation (Figure 9). Accordingly, HeLa cells undergoing this treatment also showed a significant accumulation in the G1 phase and a significant decrease in the S phase of the cell cycle, (Figure 8). Consistent with these phenotypic changes, we have shown by qPCR, that the transduction of Max* activated the transcription of the cell cycle inhibitors (p21 and p27) and repressed the transcription of CDK4 and Cyclin D1. Considering that the regulatory (up and down) effects of c-Myc on transcription are usually 2–3 fold [13], [33], the levels of activation (Figure 8B) and repression (Figure 8C) obtained here are very significant and noteworthy.

The genes affected by the transduction of Max* are *bona fide* c-Myc target genes and classically recognized to control the passage through the first checkpoint of the cell cycle and the entry into the S phase. Hence the activation p21 and p27 and the downregulation of CDK4 and Cyclin D1 are expected to lead, as observed, to an accumulation in the G1 phase and a decrease in the S phase. Consequently, an accumulation in the G2/M phase should have been observed. Although we observed such a trend, this apparent accumulation in G2 was not found to be statistically significant. On the other hand, the transduction of Max* into HeLa cells was accompanied by a significant increase in the level of cells undergoing apoptosis. This could explain, in part, the fact that we do not detect an accumulation in G2/M. Moreover, we also observed a downregulation of Cyclin B1, a key cyclin for the passage through the second checkpoint and entry into the G2 phase.

The results presented here are highly important and worthy of note in the context of c-Myc inhibition as a therapeutic tool. Moreover, they represent a step forward in the development of a therapeutic b-HLH-LZ. Indeed, whereas the systemic expression of *omomyc* - the cDNA coding for a mutated form of the c-Myc b-HLH-LZ [34] – provides an outstanding anti-tumor activity in animal models [21], [35], unlike Max*, it is not directly deliverable. Moreover, the mode of action of Omomyc *in vitro* has been recently described and shown to rely mainly on the inhibition of the transactivation of genes promoting proliferation and growth. Indeed, as shown here for Max*, Savino et al. [24] observed that Omomyc can repress the transactivation of CyclinD1, CyclinB1 and Nucleolin by c-Myc (Figure 7). However, Omomyc does not lead to the activation of Myc repressed genes as observed for Max* here (Figure 7A and B). In fact, Omomyc can repress the transcription of p21 [24]. This is due to the fact that while Omomyc can heterodimerize with Max and compete with the endogenous c-Myc/Max at activated promoters, the Omomyc/Max complex can still interact with Miz1 at repressed promoters and sustain repression. However, since Max* does not interact with Miz1 [14], its homodimeric state can compete with the endogenous c-Myc/Max (and even Max*/c-Myc heterodimer) and give rise to an activation of c-Myc repressed genes as observed here.

## Materials and Methods

HeLa cells were purchased from ATCC (American Type Culture Collection, Manassas, VA, USA). DMEM Cell culture media, serum, PBS (10X) and trypsin, were purchased from Wisent (St-Bruno, QC, Canada). Monoclonal Mouse anti-Max (H-2), anti-c-Myc (9E10), anti rat monoclonal c-Myc (3H603) and anti-rat IgG_1_•Alexa Fluor® 488, rabbit polyclonal anti-HA probe (Y-11)•Alexa-Fluor®-647 (far-red) and goat anti-rabbit•Alexa-Fluor®-488 (sc-2780) were obtained from SantaCruz Biotechnology (Santa Cruz, CA, U.S.A.). Alexa-Fluor® 488 (green) and Alexa-Fluor®-594 (red) goat anti-mouse antibodies were obtained from Molecular Probes (Burlington, ON, Canada). Western Lightning Chemiluminescence Reagent Plus was purchased from PerkinElmer Life Sciences (Woodbridge, ON, Canada). Dynasore hydrate (D7693) was obtained from Sigma-Aldrich.

### Prokaryotic expression plasmids

The human Max b-HLH-LZ corresponding cDNA (P25912, amino acids: 22–104) was first subcloned in the pET-3a expression plasmid (Novagen) by polymerase chain reaction (PCR) using pVZ1 p21max as template (kindly provided by R. N. Eisenman, Fred Hutchinson Cancer Research Center, Seattle, WA) and oligonucleotides containing 5′-*Nde*I-and 3′-*Bam*HI restriction sites. The plasmid pET3a and the PCR products were digested with the corresponding restriction enzymes before purification on agarose gel using QIAquick gel extraction Kit (Qiagen) and ligation using T4 Ligase (NewEngland Biolabs, Pickering, ON, Canada). The absence of a stop codon allowed for a 3′-GSGC extension from pET3a vector to obtain Max*-*cys* (name thereafter Max*).

For confocal microscopy, amino acids corresponding to the hemagglutinin (HA) tag (YPYDVPDYA) sequence were introduced at the C-terminus of Max*/pET3a DNA template by *QuikChange*™ Site-Directed *Mutagenesis* (Qiagen) using oligonucleotides (5′)CGT GCA CTG GAG *TAC CCA TAC GAT GTT CCA GAT TAC GCT GGA TCC* GGC and (3′)GCC GGA TCC AGC GTA ATC TGG AAC ATC GTA TGG GTA CTC CAG TGC ACG. This clone (Max*-HA) was subjected to a final *QuikChange*™ Site-Directed *Mutagenesis* to remove the last cystein using (5′)CCC ATA CGA TGT TCC AGA TTA CGC TTA AGG ATC CAC GCG G and (3′)CCG CGT GGA TCC TTA AGC GTA ATC TGG AAC ATC GTA TGG G oligonucleotides, thereby preventing unconventional protein oligomerization. Modifications were confirmed by DNA sequencing.

### Protein expression and purification

BL21 (DE3) pLysS *Escherichia coli* (Stratagene) were transformed with the b-HLH-LZ of Max* or Max*HA respectively on LB-agar containing *Ampicillin (50 µg/ml) and Chloramphenicol (34 µg/ml)* and grown at 37°C overnight. Using a single colony, a pre-culture was inoculated in 2YT medium with fresh antibiotics and grown for an additional 16 hours. 5 L of culture was then prepared using a 2% dilution from the pre-culture. The cultures were allowed to grow until turbidity reached 0.8 (D.O. at 595 nm) where protein expression was induced with 0.6 µM of IPTG (Isopropyl-ß-D-thiogalactopyranosid). After a growth period of four hours, cells were harvested by centrifugation. Soluble protein extracts were then purified by cation-exchange chromatography columns (GE Healthcare) to isolate highly pure recombinant proteins as previously described [36]. The fractions containing the Max* or the Max*HA proteins were collected, desalted on HighTrap desalting columns (GE Healthcare) using 0.05% H2O•TFA and then lyophilized with addition of 30% (^v^/_v_) acetonitrile. The proteins were solubilized in PBS buffer, pH 7.5. Protein concentrations were determined by the absorbance at 280 nm using a molar extinction coefficient factor of 2980 mol^−1^ cm^−1^ and 7450 mol^−1^ cm^−1^ for Max* and Max*HA respectively.

### Cell culture, transduction and transfection

HeLa cells (ATCC) were maintained in Dulbecco's modified Eagle's medium (DMEM) supplemented with 10% fetal bovine serum at 37°C in a humidified atmosphere containing 5% CO_2_. For confocal immunofluorescence microscopy, 35,000 HeLa cells were seeded on 0.5 inch microscopy cover glass slips and grown for 16 hours. Before treatment, cells were rinsed twice with phosphate-buffered saline (PBS) (137 mM NaCl, 3.5 mM KCl, 10 mM sodium phosphate buffer, pH 7.4). Addition of fresh media supplemented with Max* or Max*HA peptides (0–100 µM) was followed by an incubation of 5 minutes to 72 hours. The culture medium was refreshed every 48 hours. The transfection of 2 µg Dynamin1-K44A/pCB on 6-well plates seeded at 35,000 cells was done using FuGENE reagent (ROCHE). Transferrin labeled with Alexa-Fluor®-488 (50 ng/ml) and Dynasore (25–80 µM, Sigma Aldrich) was added to culture media 30 to 60 minutes preceding Max* treatments.

### Immunofluorescence

Hela cells were fixed in 3% paraformaldehyde/PBS pH 7.4, for 30 minutes, permeabilized with 0.1% Triton X–100 for 10 minutes, blocked with 10% goat serum for 30 minutes and incubated with primary antibodies for 1 hour at room temperature. After washing with PBS/1% goat serum, Alexa Fluor–488, 594 or 647-conjugated antibodies (Molecular Probes, Invitrogen) were added for an extra hour at room temperature. Cover glass slips were finally mounted on microscope slides using Permount™ Mounting medium (Fisher). The specimens were visualized using an inverted confocal laser-scanning microscope (FV1000, Olympus) equipped with a PlanApo 60×/1.42 oil immersion objective (Olympus). Olympus Fluoview software version 1.6 b was used for image acquisition and analysis.

### Proliferation and viable cell expansion assay (WST-1)

HeLa cells were incubated in 96-well plates over a period of 72 hours, increasing Max* concentration (0–50 µM). Addition of 10 µl/well of Premixed WST-1 Cell Proliferation Reagent (Clontech) was added, and the cells were incubated for an additional 90 minutes under the same conditions. Absorbance at 450 nm was measured in a multiwell plate reader.

### Cell cycle analysis-Fluorescence-activated cell scanning analysis (FACS)

Cells were analyzed with a FACScan cytometer (Becton Dickinson, Mountain View, CA) equipped with a 15 mW argon ion laser tuned at 488 nm forward and side scatter signals were used to establish the live gate to exclude debris and cell clumps. A minimum of 10,000 gated events per sample were acquired. The fluorescence of propidium iodide (PI) was collected in red channel on a linear scale detected with a 562–588 nm band pass filter. A second live gate was set using the FL3-A and FL3-W parameters of the doublet discrimination module (DDM), allowing single cell measurements. Fluorescence intensity distribution was analyzed with the CellQuest software. The percentages of cells in different phases of cell cycle were calculated by ModFit software (Verity Software House, Topsham, ME). The percentage of apoptotic cells was determined by counting the number of events below G1 phase over the total number of events.

### qPCR analysis

mRNA levels of our target genes in non-treated and treated cells were reverse transcribed and measured using SYBR green-based PCR. The qPCR experiments were carried out at the Laboratory of Functional Genomics of the Université de Sherbrooke. Primers for our gene subset were designed and validated using in-house protocols. We used a relative quantification (ΔΔCt) assay, where the target transcript level is compared to the geometric mean of a panel of 4 housekeeping genes. Data analysis was performed using the qBase analysis package [37].

## References

[1] Cole MD, Nikiforov MA (2006). Transcriptional activation by the Myc oncoprotein.. Curr Top Microbiol. Immunol.

[2] Kleine-Kohlbrecher D, Adhikary S, Eilers M (2006). Mechanisms of transcriptional repression by Myc.. Curr Top Microbiol. Immunol.

[3] Rottmann S, Luscher B (2006). The Mad side of the Max network: antagonizing the function of Myc and more.. Curr Top Microbiol. Immunol.

[4] Kime L, Wright SC (2003). Mad4 is regulated by a transcriptional repressor complex that contains Miz-1 and c-Myc.. Biochem J.

[5] Amati B, Brooks MW, Levy N, Littlewood TD, Evan GI (1993). Oncogenic activity of the c-Myc protein requires dimerization with Max.. Cell.

[6] Ayer DE, Kretzner L, Eisenman RN (1993). Mad: a heterodimeric partner for Max that antagonizes Myc transcriptional activity.. Cell.

[7] Zervos AS, Gyuris J, Brent R (1993). Mxi1, a protein that specifically interacts with Max to bind Myc-Max recognition sites.. Cell.

[8] Blackwell TK, Kretzner L, Blackwood EM, Eisenman RN, Weintraub H (1990). Sequence-specific DNA binding by the c-Myc protein.. Science.

[9] Blackwood EM, Eisenman RN (1991). Max: a helix-loop-helix zipper protein that forms a sequence-specific DNA-binding complex with Myc.. Science.

[10] Gu W, Cechova K, Tassi V, Dalla-Favera R (1993). Opposite regulation of gene transcription and cell proliferation by c-Myc and Max.. Proc Natl Acad Sci USA.

[11] Lindeman GJ, Harris AW, Bath ML, Eisenman RN, Adams JM (1995). Overexpressed max is not oncogenic and attenuates myc-induced lymphoproliferation and lymphomagenesis in transgenic mice.. Oncogene.

[12] Eilers M, Eisenman RN (2008). Myc's broad reach.. Genes & Development.

[13] Yap CS, Peterson AL, Castellani G, Sedivy JM, Neretti N (2011). Kinetic profiling of the c-Myc transcriptome and bioinformatic analysis of repressed gene promoters.. Cell Cycle.

[14] Peukert K, Staller P, Schneider A, Carmichael G, Hänel F (1997). An alternative pathway for gene regulation by Myc.. EMBO J.

[15] Staller P, Peukert K, Kiermaier A, Seoane J, Lukas J (2001). Repression of p15INK4b expression by Myc through association with Miz-1.. Nat Cell Biol.

[16] Seoane J, Pouponnot C, Staller P, Schader M, Eilers M (2001). TGFbeta influences Myc, Miz-1 and Smad to control the CDK inhibitor p15INK4b.. Nat Cell Biol.

[17] Wu S, Cetinkaya C, Munoz-Alonso MJ, Lehr von der N, Bahram F (2003). Myc represses differentiation-induced p21CIP1 expression via Miz-1-dependent interaction with the p21 core promoter.. Oncogene.

[18] Seoane J, Le H-V, Massagué J (2002). Myc suppression of the p21(Cip1) Cdk inhibitor influences the outcome of the p53 response to DNA damage.. Nature.

[19] Yang W, Shen J, Wu M, Arsura M, FitzGerald M (2001). Repression of transcription of the p27(Kip1) cyclin-dependent kinase inhibitor gene by c-Myc.. Oncogene.

[20] Bowen H (2002). c-Myc Represses and Miz-1 Activates the Murine Natural Resistance-associated Protein 1 Promoter.. Journal of Biological Chemistry.

[21] Soucek L, Whitfield J, Martins CP, Finch AJ, Murphy DJ (2008). Modelling Myc inhibition as a cancer therapy.. Nature.

[22] Soucek L, Jucker R, Panacchia L, Ricordy R, Tatò F (2002). Omomyc, a potential Myc dominant negative, enhances Myc-induced apoptosis.. Cancer Research.

[23] Soucek L, Evan GI (2010). The ups and downs of Myc biology.. Curr Opin Genet Dev.

[24] Savino M, Annibali D, Carucci N, Favuzzi E, Cole MD (2011). The action mechanism of the myc inhibitor termed omomyc may give clues on how to target myc for cancer therapy.. PLoS ONE.

[25] Noguchi H, Bonner-Weir S, Wei F-Y, Matsushita M, Matsumoto S (2005). BETA2/NeuroD protein can be transduced into cells due to an arginine- and lysine-rich sequence.. Diabetes.

[26] Kato GJ, Lee WM, Chen LL, Dang CV (1992). Max: functional domains and interaction with c-Myc.. Genes & Development.

[27] Jiao CY, Delaroche D, Burlina F, Alves ID, Chassaing G (2009). Translocation and endocytosis for cell-penetrating peptide internalization.. Journal of Biological Chemistry.

[28] Conner SD, Schmid SL (2003). Regulated portals of entry into the cell.. Nature.

[29] Song BD, Yarar D, Schmid SL (2004). An assembly-incompetent mutant establishes a requirement for dynamin self-assembly in clathrin-mediated endocytosis in vivo. Mol. Biol.. Cell.

[30] Song BD, Leonard M, Schmid SL (2004). Dynamin GTPase domain mutants that differentially affect GTP binding, GTP hydrolysis, and clathrin-mediated endocytosis.. J Biol Chem.

[31] Macia E, Ehrlich M, Massol R, Boucrot E, Brunner C (2006). Dynasore, a cell-permeable inhibitor of dynamin.. Developmental Cell.

[32] van den Berg A, Dowdy SF (2011). Protein transduction domain delivery of therapeutic macromolecules.. Curr Opin Biotechnol.

[33] O'Connell BC, Cheung AF, Simkevich CP, Tam W, Ren X (2003). A large scale genetic analysis of c-Myc-regulated gene expression patterns.. J Biol Chem.

[34] Soucek L, Helmer-Citterich M, Sacco A, Jucker R, Cesareni G (1998). Design and properties of a Myc derivative that efficiently homodimerizes.. Oncogene.

[35] Sodir NM, Swigart LB, Karnezis AN, Hanahan D, Evan GI (2011). Endogenous Myc maintains the tumor microenvironment.. Genes & Development.

[36] Jean-François N, Frédéric G, Raymund W, Benoit C, Lavigne P (2003). Improving the Thermodynamic Stability of the Leucine Zipper of Max Increases the Stability of its b-HLH-LZ:E-box complex.. J Mol Biol.

[37] Hellemans J, Mortier G, De Paepe A, Speleman F, Vandesompele J (2007). qBase relative quantification framework and software for management and automated analysis of real-time quantitative PCR data.. Genome Biol.

